# Impact on Human Health, Lifestyle, and Quality of Care After COVID-19

**DOI:** 10.3390/medicina61040630

**Published:** 2025-03-29

**Authors:** Bogdana Adriana Nasui, Codruta Alina Popescu

**Affiliations:** 1 Department of Community Medicine, Iuliu Hațieganu University of Medicine and Pharmacy, 400349 Cluj-Napoca, Romania; 2 Department of Human Sciences, Iuliu Hațieganu University of Medicine and Pharmacy, 400012 Cluj-Napoca, Romania

The COVID-19 pandemic has been an unprecedented global event in recent times, disrupting lives, economies, and healthcare systems worldwide. The ramifications of this crisis extend far beyond its acute phase, with profound and ongoing consequences for human health, lifestyle, and the quality of care. As Editors of this Special Issue, we are pleased to present the results of 16 published articles that analyze these diverse and complex impacts, offering new insights and highlighting critical areas for public health research. This Special Issue marks the beginning of a broader exploration into the pandemic’s legacy and its lessons for future healthcare challenges.

## Key Themes and Contributions

The papers published in this Special Issue bring together evidence-based analyses of the varied consequences of the COVID-19 pandemic on health outcomes and healthcare systems.

First, several studies explore the physical and functional consequences of COVID-19. Nascimento et al. [1] examine cardiac hemodynamics and functional capacity in post-COVID-19 patients, demonstrating significant reductions in exercise tolerance and systemic vascular responses. These findings underscore the lasting physical toll of the virus on survivors, and Cozma et al. [2] shed light on long COVID symptoms, revealing persistent fatigue, headache, cognitive impairments, and cardiovascular irregularities in affected individuals, which continue to diminish quality of life years after recovery. These findings reinforce the evolving nature of long COVID and the urgent need for continued research and comprehensive management strategies. Healthcare systems must adapt to support patients facing lingering effects that impact their quality of life, work capacity, and overall well-being. 

The theme of mental health and behavioral changes is addressed by Ismael et al. [3], who explore the heightened prevalence of anxiety, depression, and impulsivity post-pandemic, emphasizing the pandemic’s psychological toll on individuals across the globe, and by Popescu et al. [4], who highlight evolving mental health dynamics among medical students, pointing to the dual impact of pandemic-related stressors and the emergence of improved existing mental health support systems. As the world adapts to a new normal, prioritizing psychological well-being is very important in order to mitigate the lingering effects of COVID-19 on mental health and daily functioning.

Another group of studies investigates healthcare disruptions and surgical challenges, particularly the impact of the pandemic on surgical care pathways. Four of the published papers [5,6,7,8] collectively investigate the challenges faced in surgical care during the pandemic, ranging from delays in pancreatic cancer surgeries to shifts in pediatric and lung cancer management. These studies underscore the urgent need for resilient healthcare strategies to mitigate the impact of future health crises.

A technological and sociodemographic analysis is offered by Crnković et al. [9], who explore trends in the adoption of wearable fitness technologies among long-term care residents, revealing sociodemographic disparities in access and usage—an important consideration for future health promotion strategies.

Epidemiological insights and preventive measures are also explored. Young et al. [10] analyze foodborne disease trends in Taiwan, attributing a decrease in cases to pandemic-related protective measures. These insights underscore the importance of stringent food safety policies and consumer awareness in preventing future outbreaks and promoting public health. Similarly, Vásquez-Gómez et al. [11] demonstrate how oxygen saturation levels can predict ICU stays, offering a low-cost prognostic tool.

In the context of vaccination and public health, Fallatah et al. [12] investigate menstrual changes following COVID-19 vaccination, contributing valuable data on vaccine side effects and informing public health discussions. Meanwhile, Pitak-Arnnop et al. [13] consolidate German and international recommendations for COVID-19 booster shots, providing a comprehensive guide for healthcare providers navigating the evolving vaccination landscape.

The health of vulnerable populations is another critical area explored. Vásquez-Gómez et al. [14] highlight the increased vulnerability of patients with non-communicable diseases during the pandemic, reinforcing the need for targeted interventions to support at-risk groups. The findings show links between non-communicable diseases (NCDs), physical inactivity, and worsened COVID-19 outcomes. Roșioară et al. [15] examine health education and behavioral trends among Romanian youth, offering insights into how public health initiatives can address disparities in healthcare access and outcomes. Improving nutrition, immunization rates, and access to medical services are essential areas for enhancing the health of children and young people in Romania.

Finally, this Special Issue addresses workplace dynamics in healthcare with Surawattanasakul et al. [16], who investigate presenteeism among intern physicians during the pandemic, highlighting the prevalence and associated factors such as resource scarcity and intense workloads. This study underscores the importance of workforce well-being and effective resource allocation in maintaining healthcare performance during crises.

Together, these contributions offer a comprehensive view of the pandemic’s impact and point to key priorities for recovery, resilience, and future preparedness. The diversity of topics covered in this Special Issue reflects the complexity of COVID-19’s aftermath and the resilience of the global scientific community in addressing its challenges. From advancing our understanding of long COVID to improving mental health resources, these studies lay the groundwork for future innovations in healthcare and public policy.

This Special Issue remains open for new submissions as we continue to explore the far-reaching consequences of the pandemic. We encourage researchers to contribute original studies, reviews, and innovative solutions that advance our understanding of post-pandemic health and care.

We extend our gratitude to the authors, reviewers, and editorial team for their invaluable contributions to this Special Issue. Together, we are shaping a comprehensive narrative of the pandemic’s enduring legacy and especially its lessons for global health.

## References

[1] Nascimento E.P.d., Nascimento L.F.E.d., Castro L.d.F., Barros V.C.d., Bandeira E.R.P., Wanderley e Lima T.B., Otto-Yáñez M., Fregonezi G.A.d.F., Resqueti V.R. (2025). Cardiac Hemodynamics, Tissue Oxygenation, and Functional Capacity in Post-COVID-19 Patients. Medicina.

[2] Cozma A., Sitar-Tăut A.-V., Orășan O.H., Leucuța D.C., Pocol T.-C., Sălăgean O., Crișan C., Sporiș N.-D., Lazar A.-L., Mălinescu T.-V. (2024). The Impact of Long COVID on the Quality of Life. Medicina.

[3] Ismael M.S., Elgendy M.O., Binsaleh A.Y., Saleh A., Abdelrahim M.E.A., Osama H. (2024). Impulsivity and Its Association with Depression and Anxiety in the Normal Egyptian Population Post COVID-19 Pandemic. Medicina.

[4] Popescu C.A., Tegzeșiu A.M., Suciu S.M., Covaliu B.F., Armean S.M., Uță T.A., Sîrbu A.C. (2023). Evolving Mental Health Dynamics among Medical Students amid COVID-19: A Comparative Analysis of Stress, Depression, and Alcohol Use among Medical Students. Medicina.

[5] Puia A., Feier C.V.I., Gaborean V., Bodea R., Graur F., Zaharie F., Al-Hajjar N., Puia I.C. (2024). Changes in Pancreatic Cancer Management and Surgical Treatment During the COVID-19 Pandemic. Medicina.

[6] Feier C.V.I., Muntean C., Gaborean V., Vonica R.C., Faur A.M., Murariu M.-S., Olariu S. (2024). Surgical Challenges During the COVID-19 Crisis: A Comparative Study of Inguinal Hernia Treatment in Romania. Medicina.

[7] Jukić M., Tokić P., Elezović Baloević S., Pogorelić Z. (2024). Challenges and Solutions during the COVID-19 Pandemic: Hospitalization and Performance in Elective Pediatric Surgeries. Medicina.

[8] Cozma G.V., Muntean C., Faur A.M., Gaborean V., Petrache I.A., Feier C.V.I. (2024). Unveiling the Effects of the COVID-19 Pandemic on Lung Cancer Surgery. Medicina.

[9] Crnković I., Lončarek K., Tomasović Mrčela N., Železnik D., Vlahović T. (2025). Relationship Between the Use of Fitness Trackers and Smartwatches for Monitoring Physical Activity and the Sociodemographic Characteristics of Long-Term Care Residents During the COVID-19 Lockdown. Medicina.

[10] Yang Y.-L., Chen C.-C., Chin P.-W., Cheng C.-G., Cheng C.-A. (2024). Impact of Foodborne Disease in Taiwan during the COVID-19 Pandemic. Medicina.

[11] Vásquez-Gómez J., Gutierrez-Gutierrez L., Miranda-Cuevas P., Ríos-Florez L., Casas-Condori L., Gumiel M., Castillo-Retamal M. (2024). O_2_ Saturation Predicted the ICU Stay of COVID-19 Patients in a Hospital at Altitude: A Low-Cost Tool for Post-Pandemic. Medicina.

[12] Fallatah N.I., Alrehaili B.O., Alsulami S.S., Al-Zalabani A.H. (2024). Menstrual Changes Following COVID-19 Vaccination: A Cross-Sectional Study. Medicina.

[13] Pitak-Arnnop P., Ngamskulrungroj P., Mahanonda N., Auychai P., Frech B., Shavlokhova V., Stoll C. (2024). Current German Recommendations and International Research on the Use of COVID-19 Boosters among Health Care Providers in 2024: A Narrative Review. Medicina.

[14] Vásquez-Gómez J.A., Saracini C. (2024). Insights from Chilean NCDs Hospitalization Data during COVID-19. Medicina.

[15] Roșioară A.-I., Năsui B.A., Ciuciuc N., Sîrbu D.M., Curșeu D., Pop A.L., Popescu C.A., Popa M. (2024). Status of Healthy Choices, Attitudes and Health Education of Children and Young People in Romania—A Literature Review. Medicina.

[16] Surawattanasakul V., Kiratipaisarl W., Siviroj P. (2024). Association between Presenteeism, Associated Factors, and Outcomes among Intern Physicians in Public Hospitals during the COVID-19 Pandemic: A Cross-Sectional Study. Medicina.

